# Spontaneous Pneumomediastinum in COVID-19 Pneumonia

**DOI:** 10.7759/cureus.16129

**Published:** 2021-07-02

**Authors:** Gautam Jesrani, Samiksha Gupta, Vivek Kumar, Monica Gupta, Yajur Arya

**Affiliations:** 1 Department of General Medicine, Government Medical College and Hospital, Chandigarh, IND

**Keywords:** covid-19, spontaneous pneumomediastinum, pneumonia, mechanical ventilation, ct scan

## Abstract

Spontaneous pneumomediastinum (SPM) is the collection of air within the mediastinal cavity, which is commonly described in the literature for mechanical ventilation and perforation of hollow viscera. Coronavirus disease 2019 (COVID-19) is a rare but salient etiology of this complication in the current pandemic. Here, we are narrating a case of a 46-year-old male, in whom COVID-19 pneumonia was complicated by SPM. The complication was identified on chest computed tomography (CT) and was managed conservatively, leading to a favorable outcome. SPM has undemanding management, but timely identification and appropriate treatment institution are crucial in this milieu. A literature search revealed similar cases of SPM in COVID-19 with different outcomes and the important ones are included in this report.

## Introduction

Coronavirus disease 2019 (COVID-19) is the culprit of the current pandemic, which originated from Wuhan city, China. It is a viral disease, with rapid spread and having expeditiously evolving complication spectrum. It majorly involves the respiratory system, but extra-pulmonary involvement and complications are also being reported. Spontaneous pneumomediastinum (SPM) is one of these rare complications of COVID-19, which is becoming increasingly recognized. SPM is the air entrapment in the mediastinum without trauma or iatrogenic manipulation, primarily due to rupture of distended or diseased alveoli [1]. Most of the described literature for SPM is for mechanical ventilation, penetrating trauma, hollow organ rupture, and surgical procedures.

## Case presentation

A 46-year-old man presented with a chief complaint of breathlessness of six days to the emergency department. The severity of breathlessness increased in the last 24 hours, due to which patient came to the hospital. He also gave a history of low-grade fever, two days before the onset of breathlessness. There was no history of chest pain, palpitation, or lower limb swelling and the patient denied smoking addiction. He had no other preceding chronic illnesses.

**Figure 1:**
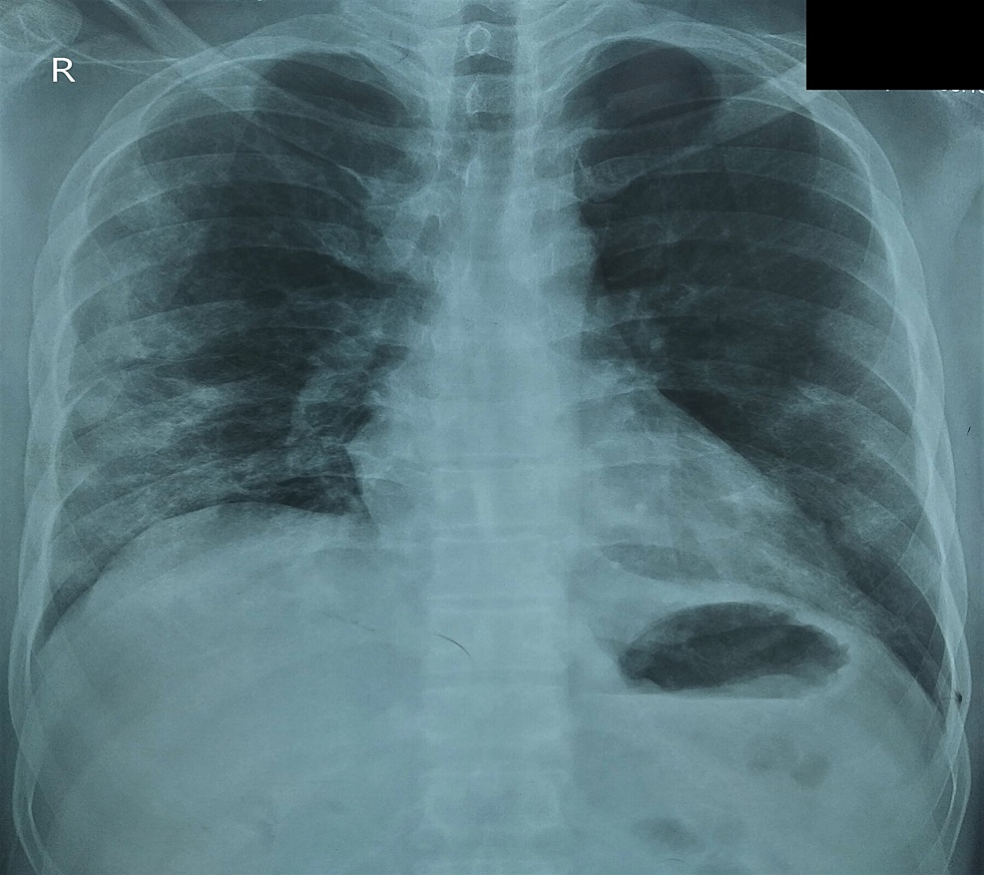
Chest x-ray posteroanterior view demonstrating bilateral (right more than left) peripheral patchy opacities.

On presentation, the patient had an oxygen saturation of 81% under room air, which raised to 95% with a non-rebreather mask and oxygen supplementation at 16 l/min. His blood pressure was 118/84 mmHg and capillary blood glucose was 89 mg/dl. A systemic examination revealed active use of the accessory muscles of respiration and a respiratory rate of 28/min. Additionally, auscultation of the chest demonstrated coarse crepitations over bilateral lung fields, but the rest of the systemic examination divulged no abnormality. Chest x-ray of the patient depicted bilateral, predominantly peripheral, patchy consolidation, and electrocardiogram was showing sinus tachycardia with a heart rate of 108/min (Figure 1). This raised the suspicion of COVID-19 infection and subsequently the patient was found to be positive for the infection by reverse transcription-polymerase chain reaction (RT-PCR). He was started on standard treatment of COVID-19, which included intravenous dexamethasone (6 mg twice a day) and subcutaneous low molecular weight heparin (60 mg twice a day).

**Figure 2:**
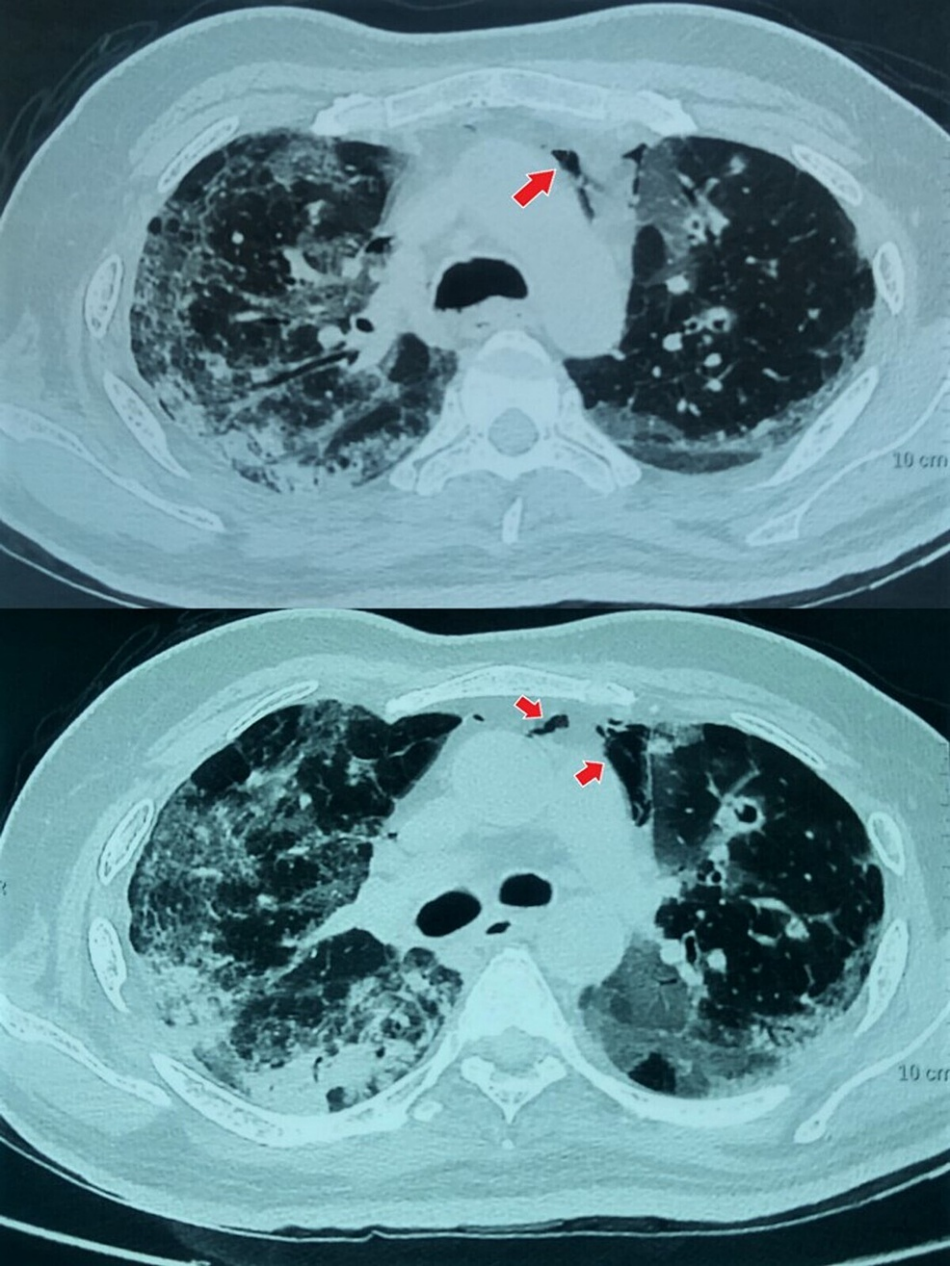
HRCT of the chest demonstrating bilateral ground-glass opacities in lung parenchyma with air foci in the mediastinum (red arrows). HRCT: high-resolution computed tomography

On additional evaluation, the patient had an elevated leukocyte count (16.5 × 10^9^/l) with 93% neutrophils and unremarkable hemoglobin level, platelet count, renal and liver function tests. Inflammatory markers, namely C-reactive protein (134 mg/dl, normal < 5), serum ferritin (748 ng/dl, normal 22-322) and D-dimer levels (2.7 µg/dl, normal < 0.5) were invariably high. Arterial blood gas analysis expressed type 1 respiratory failure and metabolic acidosis (high blood lactate levels). Further, the patient underwent high-resolution computed tomography (HRCT) of the chest, which described peripheral ground-glass opacities in bilateral lung fields, predominantly involving sub-pleural regions. Surprisingly, the CT also demonstrated a small amount of air in the mediastinum, especially behind the sternum, which was in favor of SPM, as the patient was never intubated nor had any chest trauma (Figure 2). CT severity index was 19/25 and there was no concurrent pneumothorax, pneumopericardium, or pleural effusion. So, the patient was managed conservatively with bed rest, stool softeners, and advised to avoid straining. In his 35 days hospital isolation, the patient had a gradual recovery in terms of hypoxia and was maintaining oxygen saturation of 94% with nasal cannula oxygen at 2 l/min. Subsequently, he was discharged on continuous domiciliary oxygen therapy and advised to close follow-up with the respiratory department.

## Discussion

SPM is the dissemination of air in the mediastinal cavity, which is mainly due to endotracheal intubation and positive pressure ventilation, a surgical procedure involving bronchus or esophagus, and rarely due to gas-producing infections. Recently, COVID-19 infection is confronted with this complication, which can further deteriorate the patient. The current literature search yields scarce case reports of this complication and one research by Eperjesiova et al. reported a 0.72% incidence of spontaneous air leak in COVID-19 cases (Table 1) [2-7].

**Table 1:** Various studies describing spontaneous pneumomediastinum in COVID-19 pneumonia patients. SPM: spontaneous pneumomediastinum; COVID-19: coronavirus disease 2019

Name of the study	Type of the study	Number of patients with SPM	Management/intervention	Outcome
Eperjesiova et al. [2]	Case series	5	Conservative in 4 patients, intercostal drainage in 1 patient for concurrent pneumothorax	Recovered- 4 Expired- 1
Agrawal et al. [3]	Case series	4	Conservative in 3 patients, intercostal drainage in 1 patient for concurrent pneumothorax	Recovered-2 Expired- 2
Diaz et al. [4]	Case series	3	Conservative for 2 patients, intercostal drainage in 1 patient for concurrent pneumothorax	All recovered and discharged
Kolani et al. [5]	Case report	1	Conservative	Recovered and discharged
Mimouni et al. [6]	Case report	1	Conservative	Recovered and discharged
Mohan et al. [7]	Clinical images	1	Conservative	Recovered and discharged

The proposed mechanism of SPM includes pressure imbalance between the alveoli and the surrounding interstitium. Factors leading to raised alveoli pressure are strained defecation, childbirth, excessive and forceful coughing, sneezing, or vomiting [8]. Moreover, infection with viruses like influenza and COVID-19 mutilates the types I and II pneumocytes, which leads to the alveolar membrane disintegration and air spread inside the mediastinum [9].

Presentation of SPM is not different from COVID-19 symptoms, making it impossible to identify on clinical ground. The most commonly reported symptom is retrosternal pain with radiation to the back or neck, followed by cough and dyspnea [10]. SPM can be associated with simultaneous pneumothorax or pneumopericardium, which is the air collection inside the pleural and pericardial space respectively.

Being an underdiagnosed condition, SPM suspected patients should undergo mandatory imaging modalities and ultrasound of the chest can be performed in emergency circumstances. Moderate or large SPM can be identified on chest x-ray but mild and posteriorly located air foci are invariable missed on conventional chest radiograph. Imaging interventions like CT chest is the most useful and widely available modality to identify this complication and can discover the cause in most cases. Certain radiological signs can direct the diagnosis of SPM and include thymic sail sign (thymus elevation due to air), ring sign (air around vessel), double bronchial wall sign, and continuous diaphragm outline sign [10].

Recognition of the triggering factor is crucial, as specific management is directed by the particular etiology. General measures in the treatment of SPM include bed rest, avoiding straining, coughing, and physical activity, along with the adequate analgesic institution. Likewise, oxygen administration can be considered in such patients to facilitate free air absorption [10]. All these measures are useful in mild cases or a small amount of air entrapment, but not all cases are fortunate as SPM is also known to have swift deterioration. In cases where air collection in the mediastinum is gross, rapid, and compressing the vital structures, the prognosis and outcome may be bleak. The management of these malignant scenarios comprises thoracotomy or video-assisted thoracoscopic surgery to decompress the mediastinum [11]. An active search of the ongoing air leak is also warranted in these situations to halt the progression.

The outcome of SPM is usually favorable, provided timely care and early intervention, whenever required. Mild and uncomplicated cases can demonstrate spontaneous resolution within two months, but literature also has reported recurrence of SPM [10,12]. Having a paucity of research in SPM with COVID-19, no current management guidelines are available, but conservative management and appropriate oxygen addition have elucidated the substantial outcome in numerous observations.

## Conclusions

COVID-19 is a rare etiology of SPM but in the current pandemic scenario, the virus is a paramount element for this complication development. The presentation of both viral pneumonia and SPM can be identical, but the presence of SPM can formulate the underlying disease grave. It should be suspected in cases where there is an unexpected delay in the recovery and difficult weaning off from oxygen support. CT chest is the most informative and widely used investigation in COVID-19, which can also point out the features of SPM. A treating clinician should be thoughtful of this complexity, as missing out can expand the hospital stay and unnecessary institution of COVID-19 medications.

## References

[1] Chu CM, Leung YY, Hui JY (2004). Spontaneous pneumomediastinum in patients with severe acute respiratory syndrome. Eur Respir J.

[2] Eperjesiova B, Hart E, Shokr M, Sinha P, Ferguson GT (2020). Spontaneous pneumomediastinum/pneumothorax in patients with COVID-19. Cureus.

[3] Agrawal A, Sen KK, Satapathy G, Sethi HS, Sharawat A, Reddy DS (2021). Spontaneous pneumomediastinum, pneumothorax and subcutaneous emphysema in COVID-19 patients—a case series. Egypt J Radiol Nucl Med.

[4] Diaz A, Patel D, Sayedy N, Anjum F (2021). COVID-19 and spontaneous pneumomediastinum: a case series. Heart Lung.

[5] Kolani S, Houari N, Haloua M (2020). Spontaneous pneumomediastinum occurring in the SARS-COV-2 infection. IDCases.

[6] Mimouni H, Diyas S, Ouachaou J (2020). Spontaneous pneumomediastinum associated with COVID-19 pneumonia. Case Rep Med.

[7] Mohan V, Tauseen RA (2020). Spontaneous pneumomediastinum in COVID-19. BMJ Case Rep.

[8] Newcomb AE, Clarke CP (2005). Spontaneous pneumomediastinum: a benign curiosity or a significant problem?. Chest.

[9] Gralinski LE, Baric RS (2015). Molecular pathology of emerging coronavirus infections. J Pathol.

[10] Kouritas VK, Papagiannopoulos K, Lazaridis G (2015). Pneumomediastinum. J Thorac Dis.

[11] Perna V, Vilà E, Guelbenzu JJ, Amat I (2010). Pneumomediastinum: is this really a benign entity? When it can be considered as spontaneous? Our experience in 47 adult patients. Eur J Cardiothorac Surg.

[12] Gerazounis M, Athanassiadi K, Kalantzi N, Moustardas M (2003). Spontaneous pneumomediastinum: a rare benign entity. J Thorac Cardiovasc Surg.

